# Large-scale meta- and cross-trait analyses uncover shared genetic risk factors for IBS and psychiatric disorders

**DOI:** 10.3389/fpsyt.2026.1795532

**Published:** 2026-05-14

**Authors:** Weiyi Ye, Huanxin Ding, Bichen Peng, Xianjin Wang, Yugang Cheng, Guangyong Zhang, Hongwei Yu, Kalim Ullah, Xiao Chang, Xiaoyan Wang, Zhiyu Wang

**Affiliations:** 1 College of Medical Information and Artificial Intelligence, Shandong First Medical University, Jinan, China; 2 Department of General Surgery, The First Affiliated Hospital of Shandong First Medical University and Shandong Provincial Qianfoshan Hospital, Jinan, China; 3 West China Second University Hospital, Sichuan University and West China Women’s and Children’s Hospital, Chengdu, China; 4 First Affiliated Hospital of Shandong First Medical University, Biomedical Sciences College & Shandong Medicinal Biotechnology Centre, Shandong First Medical University, Jinan, China

**Keywords:** eQTL, genetic correlation, gut-brain axis, irritable bowel syndrome, mQTL, MTAG, pleiotropy, psychiatric comorbidity

## Abstract

**Introduction:**

Irritable bowel syndrome (IBS) is a common gut-brain axis disorder characterized by abdominal pain and altered bowel habits, and it shows high comorbidity with psychiatric disorders. However, the shared genetic mechanisms underlying these associations remain incompletely understood.

**Methods:**

We performed a large-scale meta-analysis of IBS in individuals of European ancestry by integrating genome-wide association study (GWAS) summary statistics from the UK Biobank, Bellygenes, and the Million Veteran Program (MVP), thereby increasing statistical power to detect novel IBS loci. We further conducted global genetic correlation analyses with psychiatric traits, followed by multi-trait analysis of GWAS (MTAG) and conditional false discovery rate (condFDR) analyses to identify pleiotropic loci. Transcriptomic, methylomic, and expression quantitative trait locus (eQTL) data were integrated to explore potential regulatory mechanisms.

**Results:**

The meta-analysis identified up to ten previously unreported IBS loci, several of which were supported by colonic and brain eQTL effects. Global genetic correlation analyses confirmed substantial genetic overlap between IBS and psychiatric traits, particularly major depressive disorder and neuroticism. MTAG and condFDR analyses uncovered more than 100 pleiotropic loci, including signals at *SORCS1, SLC35D1, COA1*, and *TLE1*. Integrative analyses of transcriptome- and methylome-wide data highlighted regulatory mechanisms spanning colonic, immune, and neuronal tissues, supporting neuro-immune crosstalk and mitochondrial involvement.

**Discussion:**

Our findings provide a comprehensive genetic characterization of IBS, refine its heritable basis, reveal pleiotropic links with psychiatric disorders, and implicate molecular pathways across the gut-brain axis. These results advance mechanistic understanding of IBS and may inform future therapeutic development for IBS and its psychiatric comorbidities.

## 1 Introduction

Irritable Bowel Syndrome (IBS) is a common and symptomatically complex functional gastrointestinal disorder—affecting between 5% and 10% of the general population globally (1, 2)—characterized by recurrent abdominal pain associated with alterations in bowel habits, which significantly impairs patients’ quality of life and imposes a substantial healthcare and societal burden (1, 2). In recent years, the “gut-brain axis” has emerged as a central framework for understanding the pathophysiology of IBS. This model posits the existence of bidirectional communication pathways involving the central nervous system (CNS), autonomic nervous system (ANS), enteric nervous system (ENS), immune system, and gut microbiota, with IBS arising from dysregulation within these interconnected systems (3–8). Epidemiologically, IBS exhibits high comorbidity with psychiatric conditions (e.g., anxiety and depression) and neuroticism (NE), a personality trait associated with vulnerability to psychiatric disorders and frequently analyzed alongside psychiatric phenotypes in genetic studies. This overlap suggests potential shared biological underpinnings (9). However, the shared genetic architecture driving these associations and the specific molecular and cellular mechanisms involved remain unknown.

Genome-wide association studies (GWAS) have markedly advanced the genetic dissection of complex diseases (10, 11). The landmark study involving a meta-analysis of the Bellygenes initiative and the UK Biobank (UKB) cohorts provided a large-scale genetic characterization of IBS, confirming its detectable heritability and identifying six genome-wide significant loci near genes including *NCAM1*, *CADM2*, *PHF2/FAM120A*, *DOCK9*, *CKAP2/TPTE2P3*, and *BAG6* (12). Nevertheless, due to the high phenotypic heterogeneity and relatively low single nucleotide polymorphism (SNP)-based heritability of IBS, many genetic signals with weak and distributed effects likely remain undetected as a result of limited statistical power. More importantly, previous studies lack systematic, multi-omics integration elucidating how risk variants influence gene expression in tissues relevant to the gut-brain axis—such as the colon, brain regions, and immune system—and across specific cell types (13–15). Furthermore, although large-scale GWAS from the Psychiatric Genomics Consortium (PGC) (16) provide a solid foundation for investigating genetic correlations with psychiatric disorders and neuroticism (e.g., major depressive disorder [MDD], NE, obsessive-compulsive disorder [OCD], post-traumatic stress disorder [PTSD], attention-deficit/hyperactivity disorder [ADHD]), the extent of genetic sharing between IBS and these traits—both genome-wide and at local regions—as well as the existence of robust pleiotropic hubs, has not been comprehensively delineated. The recent release of large IBS cohorts from the VA Million Veteran Program (MVP) in 2024 presents a timely opportunity to enhance the statistical power of genetic discovery in IBS and to enable cross-trait analyses coupled with multi-omics functional annotation (17).

To address current gaps in IBS genetics, we conducted a large-scale inverse-variance weighted meta-analysis by integrating the latest Million Veteran Program (MVP) cohorts with the largest available UKB-Bellygenes IBS dataset. This significantly improves statistical power to map IBS susceptibility loci and dissect shared mechanisms with psychiatric traits. Our three-tiered strategy involves: (i) discovery of novel and refined risk loci through meta-analysis; (ii) identification of pleiotropic hubs using a dual analytical framework—MTAG and (conj)condFDR—together with global and local genetic correlation analyses (LDSC and ρ-HESS); and (iii) multi-omics integration via TWAS (GTEx v8, MashR), tissue- and cell-type specific eQTLs, and mQTL profiling to link variants to regulatory mechanisms across the gut-brain-immune axis. Compared to previous studies, our approach introduces key innovations: increased statistical power, dual-scale correlation mapping, robust pleiotropy detection that mitigates LD and sample overlap confounding, and a multi-layered functional annotation framework. Collectively, these advances lay the groundwork for mechanism-driven therapeutic development and stratified care in IBS.

## 2 Methods

### 2.1 GWAS data

We compiled GWAS summary statistics from individuals of European ancestry inferred from genetic data from publicly available sources. First, we obtained the largest-to-date GWAS for irritable bowel syndrome (IBS), termed IBS-UKB/Bellygenes (53,400 cases and 433,201 controls). This dataset was derived from a prior meta-analysis (12) that integrated two independent cohorts: the UK Biobank (UKB; 40,548 cases and 293,220) and the Bellygenes initiative (12,852 cases and 139,981 controls). Specifically, cases were defined using a combination of Rome III symptom criteria, clinician-verified diagnoses, and hospital-linked ICD-10 codes across both cohorts. Controls were defined as digestively healthy individuals, with strict exclusion criteria applied to both cases and controls to remove participants with organic gastrointestinal diseases, such as inflammatory bowel disease or celiac disease, thereby preventing signal contamination. We also incorporated two recently released IBS datasets from the VA Million Veteran Program: IBS-MVP1 (survey-based phenotype; 15,794 cases and 299,874 controls) (17) and IBS-MVP2 (electronic health record diagnosis using PheCode 564.1; 14,867 cases and 427,355 controls) (17). Controls for IBS-MVP1 were defined as participants who did not report an IBS diagnosis in the enrollment survey, while IBS-MVP2 controls were EHR-derived non-cases lacking the IBS PheCode. The larger control pool in IBS-MVP2 reflects the near-universal availability of clinical records, whereas IBS-MVP1 is limited to participants who responded to the specific enrollment questionnaire.

GWAS data for ten additional psychiatric disorders were obtained from the Psychiatric Genomics Consortium (PGC): anorexia nervosa (AN; 16,992 cases and 55,525 controls) (18); attention-deficit/hyperactivity disorder (ADHD; 38,691 cases and 186,843 controls) (19), anxiety disorders (ANX; 7,016 cases and 14,745 controls) (20), autism spectrum disorder (ASD; 18,381 cases and 27,969 controls) (21), bipolar disorder (BD; 41,917 cases and 371,549 controls) (22), major depressive disorder (MDD; 170,756 cases and 500,199 controls) (23), obsessive-compulsive disorder (OCD; 53,660 cases, 2,044,417 controls) (24), panic disorder (PD; 2,147 cases and 7,760 controls) (25), post-traumatic stress disorder (PTSD; 29,556 cases and 166,145 controls) (26), and schizophrenia (SCZ; 74,776 cases and 101,023 controls) (27). Across PGC cohorts, controls were generally defined as cohort-specific non-cases, often screened for the absence of lifetime psychiatric disorders. Stringent control definitions were used in specific traits, such as “super-normal” individuals for ANX and trauma-exposed non-cases for PTSD. Additionally, family-based cohorts (e.g., ASD, OCD, and SCZ) utilized pseudo-controls derived from non-transmitted parental alleles. The GWAS summary statistics for neuroticism (NE), a stable personality trait reflecting negative emotionality, were obtained from the GWAS Catalog (28), comprising data from 523,783 participants. NE was analyzed as a continuous quantitative trait across population cohorts; therefore, no binary case-control definition was applied to this dataset.

Detailed information on all GWAS datasets analyzed in this study is provided in **Supplementary Table 1**.

### 2.2 Meta-analysis between IBS-UKB/Bellygenes and IBS-MVP

Inverse-variance-weighted meta-analyses were conducted using the “-meta-analysis” function in PLINK v1.90b7 (29), combining IBS-UKB/Bellygenes separately with IBS-MVP1 and IBS-MVP2. Fixed-effect meta-analytic P-values and odds ratios (ORs) were calculated. SNPs reaching genome-wide significance in the meta-analysis (*P* < 5 × 10^−8^) with suggestive significance (*P* < 0.01) in the corresponding single-trait GWAS were prioritized.

### 2.3 eQTL annotation

Each novel significant locus was queried through the GTEx Portal (https://www.gtexportal.org/home/). The GTEx project provides eQTL data from 49 human tissues (each with a sample size greater than 70), employing standardized pipelines for gene expression quantification, covariate adjustment, and cis-eQTL mapping. Significant associations were identified using an FDR ≤ 0.05 threshold. For each variant, eQTL associations across multiple tissues were extracted, and results meeting the significance threshold of *P* < 1 × 10^−5^ were retained and compiled.

### 2.4 sc-eQTL annotation

Single-cell eQTL annotations were obtained using a similar manual lookup strategy via the scQTLbase platform (https://bioinfo.szbl.ac.cn/scQTLbase/Search/). Each SNP was individually queried to retrieve cell type-specific eQTL associations. scQTLbase is an integrated human single-cell eQTL database, covering 304 datasets, 57 cell types, and 95 cell states, encompassing approximately 16 million SNPs significantly associated with gene expression.

### 2.5 mQTL annotation

We utilized mQTL summary statistics from three published studies:1) McRae et al. (30): This study was based on the Brisbane Systems Genetics Study (n = 614) and the Lothian Birth Cohort (n = 1366), using the Illumina HumanMethylation450 array to measure methylation status, covering 485,512 DNA methylation probes. 2) Li et al. (31): This study provided cross-population (European and East Asian ancestry) cis-mQTL summary statistics, generated via inverse-variance weighted meta-analysis, with a significance threshold set at *P* < 10^−10^. The study aimed to quantify the extent of shared and heterogeneous genetic regulation across different ancestry groups. 3) Hannon et al. (32): This study included mQTL summary statistics for multiple complex traits and evaluated the extent to which mQTLs explain phenotypic variation. Each novel locus was matched against these datasets to extract relevant results, which were then compiled and organized.

### 2.6 Transcriptome-wide association study

We performed transcriptome-wide association studies (TWAS) using the MashR model (33) pre-trained on GTEx v8 data to leverage cross-tissue genetic regulation. GWAS summary statistics were imputed using the 1000 Genomes EUR reference panel via MetaXcan to maximize SNP compatibility. We analyzed all GTEx v8 tissues, prioritizing gut-brain axis relevance, including 13 brain regions, digestive tissues (colon/small intestine), liver, pancreas, adipose, and blood/immune tissues. Gene-level associations were assessed using S-PrediXcan (34, 35) for tissue-specific effects and S-MultiXcan for cross-tissue integration (condition number threshold=30). Significance was determined by integrating single-tissue p-values, cross-tissue consistency, and multi-tissue meta-analysis results.

### 2.7 Global genetic correlation analysis

To quantify the shared genetic architecture between irritable bowel syndrome (IBS) and psychiatric disorders, genetic correlation (r_g_) estimates were computed through linkage disequilibrium score regression (LDSC) (36) using publicly available GWAS summary statistics. The analysis employed precomputed linkage disequilibrium scores derived from HapMap3 SNPs, which were calculated based on European-ancestry individuals from the 1000 Genomes Project Phase 3 reference panel to ensure population-matched LD structure. Standard quality control procedures were implemented, including the exclusion of SNPs with ambiguous strand orientation, low minor allele frequency (MAF < 1%), or poor imputation quality (INFO score < 0.9). The intercept term was constrained to account for potential residual population stratification and cryptic relatedness in the input GWAS data.

### 2.8 Local genetic correlation analysis

Given that the genetic correlations estimated by LDSC aggregate information from all variants across the genome, we performed additional analyses using ρ-HESS (Heritability Estimation from Summary Statistics) to evaluate pairwise local genetic correlations between each pair of traits within 1,703 predefined LD-independent segments (37). ρ-HESS is particularly suitable for estimating local genetic correlations within each of these segments, which have an average length of 1.6 Mb. To determine statistical significance, we applied Bonferroni correction, considering p-values below 0.05/1703 as statistically significant.

### 2.9 Multi-trait analysis of GWAS

MTAG was applied to explore the genetic overlap between irritable bowel syndrome (IBS) and psychiatric traits by leveraging joint GWAS summary statistics (38). This method enhances the detection of trait-relevant loci through the integrated analysis of GWAS summary statistics across multiple traits. Compared to conventional inverse-variance-weighted meta-analysis, MTAG additionally accounts for sample overlap and incomplete genetic correlations between traits. In the initial step of MTAG, variants were filtered by excluding low-frequency single nucleotide polymorphisms (SNPs), duplicate SNPs, and those with strand ambiguity. Pairwise genetic correlations between traits were then estimated using LDSC, and these estimates were used to calibrate the variance-covariance matrix of the random-effects component. Following calibration, MTAG performed a random-effects meta-analysis to generate SNP-level summary statistics. Pleiotropic SNPs that reached genome-wide significance (*P* < 5×10^-8^) in the multi-trait analysis and suggestive significance (*P* < 0.01) in the single-trait GWAS were prioritized. Due to its complex linkage disequilibrium (LD) structure, the MHC region was treated as a single locus. Results were visualized using Circos (39).

### 2.10 Conditional false discovery rate

We applied the condFDR approach to identify loci associated with IBS by conditioning the genetic associations of IBS on each psychiatric trait. The condFDR method extends the standard FDR framework and operates within an empirical Bayesian framework, leveraging the combined power of two GWAS to enhance the discovery rate of genetic variants (40). This approach utilizes associations of single nucleotide polymorphisms (SNPs) with both the primary phenotype (IBS) and conditional phenotypes (e.g., schizophrenia) to prioritize variants that are more likely to represent true associations, even if their p-values do not reach the conventional genome-wide significance threshold (40, 41). The condFDR procedure reorders the SNP p-values from the primary phenotype (IBS) based on the strength of association in the conditional phenotypes (five psychiatric traits). As a result, IBS variants that show association with the five psychiatric traits are assigned lower condFDR estimates (40–42). Similarly, the conjFDR approach—implemented by reversing the roles of the primary and conditional phenotypes in an inverse condFDR analysis—enables the detection of SNPs associated with both the primary and conditional phenotypes. The conjFDR statistic is defined as the maximum of the two conditional FDR values. In our analysis, a threshold of 5% was applied for both conditional FDR and conjoint FDR.

## 3 Results

### 3.1 Meta-analysis of IBS using UKB-Bellygenes and MVP datasets

**Figure 1:**
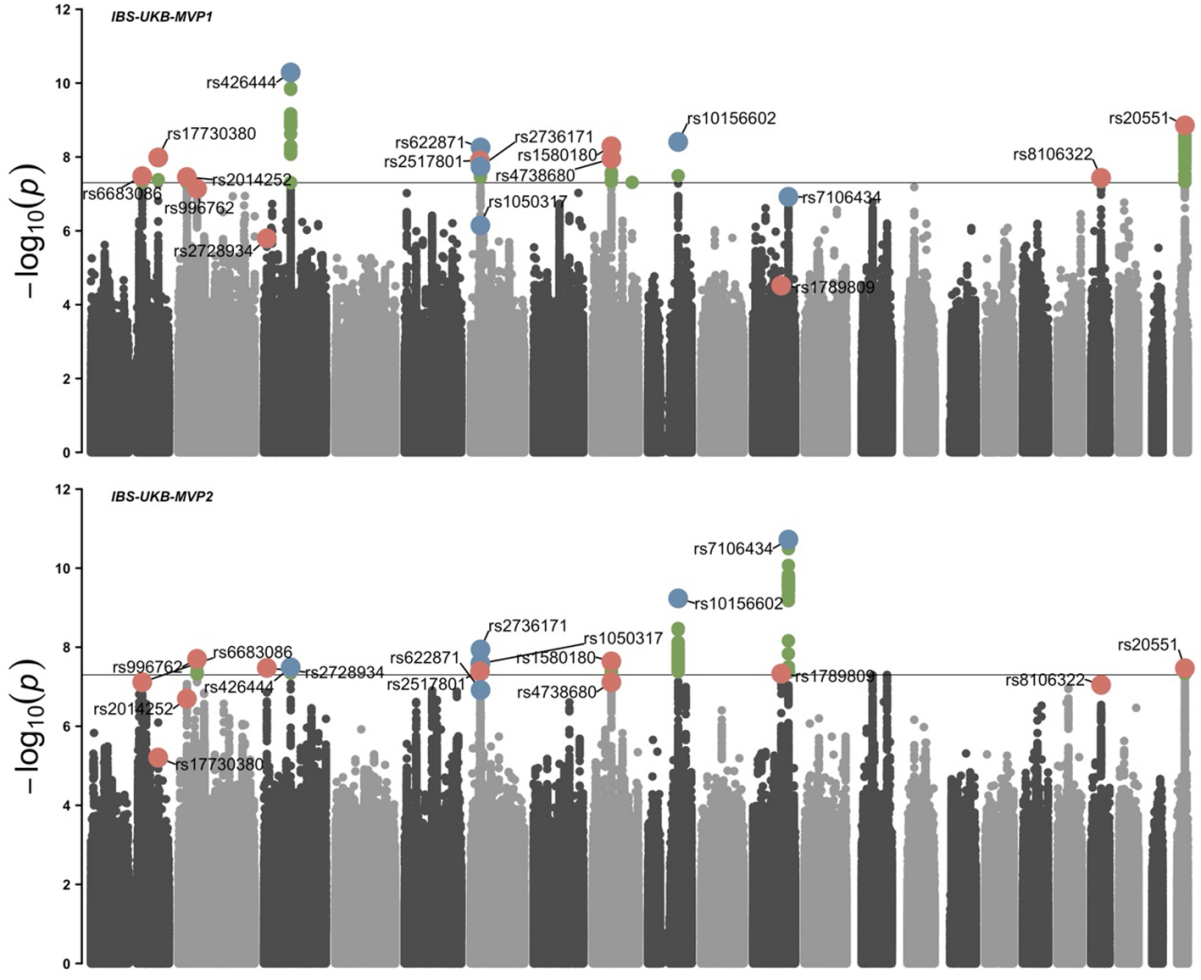
Manhattan plots of meta-analysis.Red dots represent novel lead loci that became significant after meta-analysis (previously unreported in the IBS-UKB/Bellygenes cohort), while blue dots indicate known significant loci. Green dots denote SNPs that reached genome-wide significance but are not lead SNPs in their respective loci. The black line denotes the genome-wide significance threshold of *P* < 5 × 10^-8^.

We performed two independent inverse-variance weighted meta-analyses by integrating the IBS-UKB/Bellygenes dataset with each of the two MVP cohorts separately, accounting for the partial sample overlap between IBS-MVP1 (survey-based phenotype) and IBS-MVP2 (diagnosis-based phenotype). The resulting datasets—IBS-UKB/Bellygenes-MVP1 and IBS-UKB/Bellygenes-MVP2—identified nine and ten genome-wide significant loci (*P* < 5 × 10^-8^), respectively. After excluding previously reported loci and the extended MHC region (chr6: 26–34 Mb), we detected six novel risk loci in IBS-UKB/Bellygenes-MVP1 and five in IBS-UKB/Bellygenes-MVP2 that were not genome-wide significant in the original IBS-UKB/Bellygenes dataset (**Figure 1**). All of these loci had previously shown suggestive evidence of genetic overlap between IBS and related traits, but did not reach genome-wide significance in the original IBS-UKB/Bellygenes GWAS due to limited statistical power. (**Supplementary Table 2**) (14, 40). Notably, rs1580180 (from IBS-UKB/Bellygenes-MVP1) and rs4738680 (from IBS-UKB/Bellygenes-MVP2) mapped to the same LD block (R² = 0.96), indicating convergence between the two phenotyping approaches despite differences in how IBS was defined in MVP1 (survey-based) versus MVP2 (diagnosis-based). In addition, rs20551 was robustly replicated across both meta-analyses, further supporting their potential roles in IBS susceptibility.

To explore the functional relevance of the newly identified loci from the meta-analysis, we conducted tissue- and cell-type-specific QTL annotation. Using GTEx v8 data, we identified multiple SNPs—including rs2014252 (2p23.3), rs2728934 (3p25.3), rs20551 (22q13.2), and rs1580180/rs4738680 (8q12.1)—as expression quantitative trait loci (eQTLs) in colonic tissue (**Supplementary Table 3**). Several genes near these variants, such as ATRAID, MEI1/XPNPEP3, UBXN2B, and SRGAP3, also demonstrated coordinated expression in both colon and brain, supporting the gut-brain regulatory axis in IBS pathophysiology (13, 14, 43). To further characterize cell-type specificity, we integrated single-cell eQTL datasets from peripheral blood mononuclear cells (44), human iPSC-derived midbrain neurons (45), and CD4^+^ T cells (46), revealing serotonergic and immune cell co-expression of ATRAID and XPNPEP3, with MEI1, UBXN2B, and SRGAP3 showing restricted expression in glial or progenitor subtypes (**Supplementary Table 4**). These genes align with prior findings implicating impaired serotonergic and dopaminergic signaling in IBS (47–50). Furthermore, methylation QTL (mQTL) profiling identified 81 novel associations, with rs2014252 and rs20551 showing broad regulatory effects on 16 and 55 CpGs, respectively, suggesting potential pleiotropic action via both transcriptional and epigenetic mechanisms (**Supplementary Table 5**).

### 3.2 Transcriptome-wide association study of IBS identifies gut-brain regulatory mechanisms

Using TWAS analyses (IBS-UKB/Bellygenes-MVP1/2) centered on newly identified loci, we detected convergent signals across gut, brain, and immune-related tissues, consistent with gut-brain axis involvement in IBS. The most robust and replicated association was *SUPT7L* in cultured fibroblasts (*P* ≈ 2.4-2.97 × 10^-7^). Notably, several genes showed cross-tissue patterns linking colon and brain. AGMO exhibited its strongest association in colon sigmoid (IBS-UKB/Bellygenes-MVP1, *P* = 3.94 × 10^-3^), while *TP53TG1* showed a tissue “switch,” peaking in transverse colon (IBS-UKB/Bellygenes-MVP1) and cerebellum (IBS-UKB/Bellygenes-MVP2) (*P* = 1.25 × 10–^2^ and 5.90 × 10^-3^, respectively). *SIRT3* reached significance with primary signals in brain regions (e.g., anterior cingulate; *P* = 3.38 × 10–^3^ and 6.45 × 10^-3^), and *HLA-DQA1* demonstrated strong associations with marked tissue heterogeneity across brain regions (*P* ≈ 1-2 × 10^-3^; z spanning negative to near-null). Additional brain-enriched signals included *ZNF143* (nucleus accumbens), *DLGAP1*, *KCNV2*, *PTBP1*, and *PHRF1*, while lncRNA/transcript signals at the *AARSD1*/*RP11-326C3* locus appeared across colon, blood, salivary gland, and cerebellar tissues. Immune modulators (*PDCD1LG2*, *IRF7*, *AZU1*) also showed suggestive associations, aligning with single-cell eQTL evidence in CD4^+^ T cells and neuronal/glial populations (serotonergic-like and dopaminergic neurons, oligodendrocytes). Collectively, TWAS highlights metabolic/mitochondrial (*SIRT3*) (51), lipid/membrane (*AGMO*) (52), immune-inflammatory (*HLA-DQA1*, *PDCD1LG2*, *IRF7*), and neuronal-glial regulatory nodes (*DLGAP1*) (53), supporting a multi-tissue regulatory architecture along the gut-brain axis in IBS (**Supplementary Table 6**).

### 3.3 Genetic correlations among IBS and psychiatric disorders

SNP-based heritability estimates (h²) for irritable bowel syndrome (IBS) were generally low to modest across cohorts. The highest estimate was observed in the UK Biobank (UKB; h² = 0.0247 ± 0.0015), while both MVP1 (h² = 0.0110 ± 0.0019) and MVP2 (h² = 0.0089 ± 0.0012) exhibited lower values. Meta-analytic models modestly improved precision, with heritability estimates of 0.0189 ± 0.0010 and 0.0163 ± 0.0009 for IBS-UKB/Bellygenes-MVP1 and IBS-UKB/Bellygenes-MVP2, respectively. These heritability levels are notably lower than those observed in core psychiatric conditions such as schizophrenia (SCZ, h² = 0.379), Parkinson’s disease (PD, h² = 0.308), or autism spectrum disorder (ASD, h² = 0.204), and are more comparable to traits like obsessive-compulsive disorder (OCD, h² = 0.010), bipolar disorder type II (BD-II, h² = 0.013), and post-traumatic stress disorder (PTSD, h² =0.019) (**Supplementary Table 7**).

**Figure 2:**
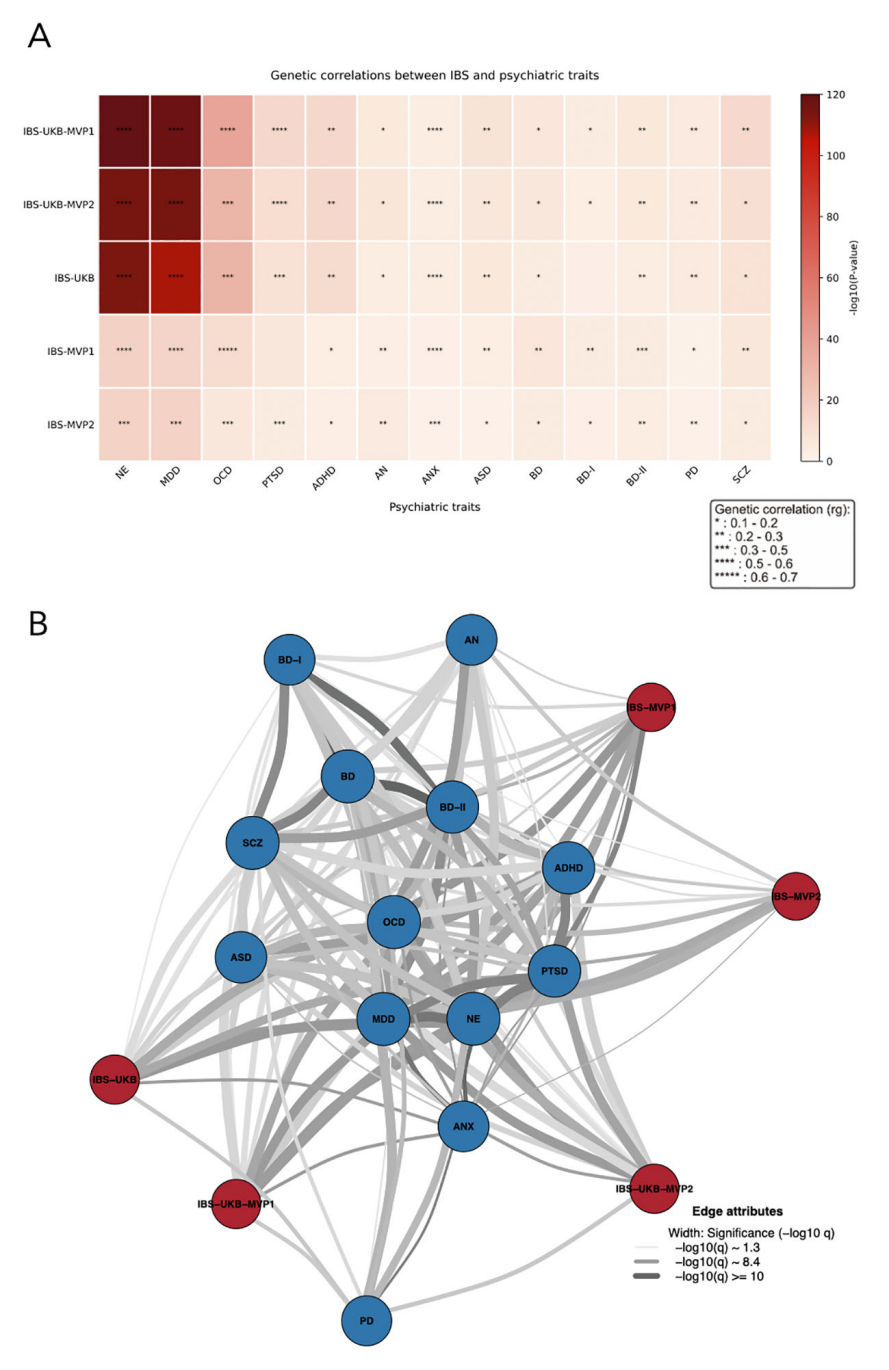
Global genetic correlations among IBSs and psychiatric traits. **(A)** Heatmap of genetic correlation estimates between IBS and psychiatric traits. The number of asterisks (∗) represents the strength of the genetic correlation. Darker red shades indicate more significant FDR values, and asterisks denote statistically significant correlations (FDR < 0.05). **(B)** Genetic correlation networks across IBSs and psychiatric traits. Each circle within the network represents a disease or trait, and edges depict significant genetic correlations (FDR < 0.05). IBS, irritable bowel syndrome; AN, anorexia nervosa; ANX, anxiety; NE, neuroticism; MDD, major depressive disorder; SCZ, schizophrenia; ADHD, attention-deficit hyperactivity disorder; BD, bipolar disorder; PTSD, post-traumatic stress disorder; ASD, autism spectrum disorder; OCD, obsessive-compulsive disorder; PD, panic disorder; UKB, UK Biobank; MVP, Million Veteran Program.

To examine shared genetic architecture between IBS and psychiatric conditions, we performed linkage disequilibrium score regression (LDSC) across all IBS GWAS datasets, using the IBS-UKB-MVP1 meta-analysis as the representative result set (the independent IBS-UKB-MVP2 meta-analysis produced highly similar findings). Strong and highly consistent positive genetic correlations were detected between IBS and multiple brain-related phenotypes, particularly NE (rg = 0.576, P = 4.89 × 10^-^¹²^5^) and MDD (rg = 0.566, P = 5.72 × 10^-^¹¹^8^), as well as OCD (rg = 0.501, P = 2.67 × 10^-^³^8^), PTSD (rg = 0.556, P = 3.02 × 10^-^¹^4^), and ADHD (rg = 0.258, P = 3.74 × 10^-^¹^4^). More moderate associations were observed for SCZ (rg = 0.206, P = 1.43 × 10^-^¹³), ASD (rg = 0.246, P = 6.89 × 10^-9^), BD (rg = 0.160, P = 1.03 × 10^-7^), BD-II (rg = 0.264, P = 1.61 × 10^-6^), and AN (rg = 0.169, P = 2.61 × 10^-6^). Additional significant positive correlations were also observed for panic disorder (PD) (rg = 0.280, P = 1.64 × 10^-5^), BD-I (rg = 0.116, P = 1.00 × 10^-4^), and ANX (rg = 0.563, P = 2.00 × 10^-4^), although these estimates were less precise. Compared with the meta-analyses involving UKB/Bellygenes and MVP, the single-cohort MVP analyses showed larger standard errors but maintained consistent directions of effect (**Figure 2A**; **Supplementary Table 8**). To visualize the broader architecture of shared liability, we constructed a correlation network based on pairwise genetic correlations (**Supplementary Table 8**; **Figure 2B**). The resulting network revealed that core affective and compulsive disorders—namely MDD, ANX, OCD, and NE—formed a tightly connected module. This cluster further linked to neurodevelopmental and psychotic traits such as SCZ, PTSD, and ADHD. Strikingly, multiple IBS datasets were positioned in close proximity to this central psychiatric cluster, underscoring a shared gut-brain axis genetic architecture.

**Figure 3:**
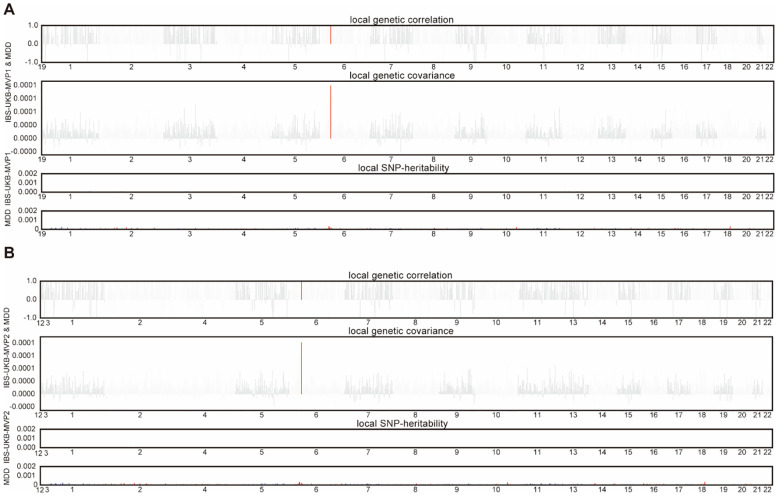
Local genetic correlations between IBS-UKB/Bellygenes-MVP1/2 and MDD. **(A)** Manhattan plot showing the estimates of local genetic correlation, genetic covariance, and SNP heritability between IBS-UKB/Bellygenes-MVP1 and MDD. **(B)** Manhattan plot showing the estimates of local genetic correlation, genetic covariance, and SNP heritability between IBS-UKB/Bellygenes-MVP2 and MDD. Red bars represent loci showing significant local genetic correlation after multiple testing adjustment. IBS, irritable bowel syndrome; MDD, major depressive disorder; UKB, UK Biobank; MVP, Million Veteran Program.

To localize genomic regions driving these shared effects, we next performed local genetic correlation analysis. After correcting for multiple testing, significant regional correlations were observed in the extended major histocompatibility complex (MHC) region, particularly between IBS-UKB/Bellygenes-MVP1 and MDD (**Figure 3A**), and similarly between IBS-UKB/Bellygenes-MVP2 and MDD (**Figure 3B**). These results highlight potential immunogenetic mechanisms contributing to IBS-psychiatric comorbidity.

### 3.4 MTAG uncovers novel pleiotropic loci between irritable bowel syndrome and psychiatric disorders

**Figure 4:**
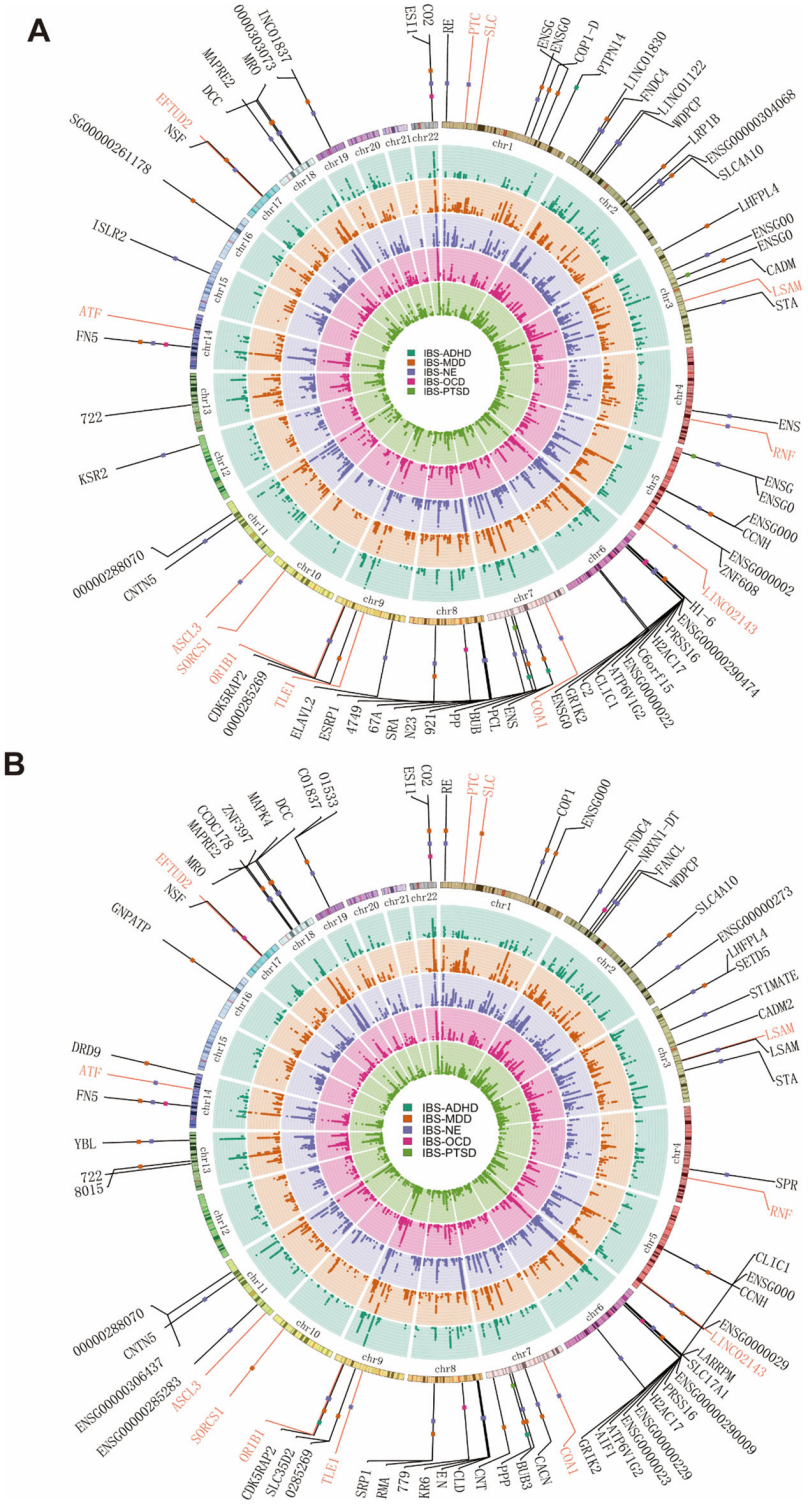
Overview of pleiotropic loci identified through multi-trait analyses of IBS-UKB/Bellygenes-MVP1 and IBS-UKB/Bellygenes-MVP2.**(A)** The upper circle illustrates pleiotropic loci detected by MTAG analysis between IBS-UKB/Bellygenes-MVP1 and psychiatric traits, while the lower circle **(B)** shows corresponding results for IBS-UKB/Bellygenes-MVP2. Orange lines denote novel pleiotropic loci. Dots along the lines are colored according to the specific trait analyzed in the meta-analysis. IBS, irritable bowel syndrome; NE, neuroticism; MDD, major depressive disorder; ADHD, attention-deficit hyperactivity disorder; PTSD, post-traumatic stress disorder; OCD, obsessive-compulsive disorder.

Building upon the enhanced genetic correlation between IBS and psychiatric disorders achieved through meta-analysis of IBS-UKB/Bellygenes and IBS-MVP, we sought to further identify their shared pleiotropic loci. Given the well-established strong brain–gut axis association in IBS (**Figure 1**), we first applied multi-trait analysis of GWAS (MTAG) to pair IBS with five psychiatric traits showing robust genetic correlations with IBS via LDSC (MDD, NE, OCD, PTSD, and ADHD). After excluding the MHC region, we identified 124 independent loci, 83 of which were novel to the original IBS-UKB/Bellygenes (**Figure 4**)—though many had been reported in subsequent joint analyses (14, 15). As expected, most novel loci were detected in MTAG analyses pairing IBS with MDD and neuroticism. Notably, several established loci reside near genes implicated in brain development and synaptic function, such as *CADM2* (3p12.1) and *NCAM1* (11q23.2). However, the MTAG analysis revealed additional novel loci linked to neuronal development, including *RERE* (1p36.23), *SLC4A10* (2q24.2), *SETD5* (3p25.3), *CACNA2D1* (7q21.11), *CDK5RAP2* (9q33.2), *PPP1R3A* (7q31.1), and *LRFN5* (14q21.1). Furthermore, several novel pleiotropic loci were consistently identified across multiple MTAG analyses of IBS and various psychiatric disorders, including *CCNH*, *SLC4A10*, *DCC*, and *MRO* (**Supplementary Tables 9**-**13**). Importantly, we also detected several truly novel loci—unreported in either IBS-UKB/Bellygenes or prior joint analyses—such as three in the analysis of IBS-UKB/Bellygenes-MVP with MDD: *SLC35D1* (1p31.3), *RNU7-66P* (6q12), and *SORCS1* (10q25.1) (**Supplementary Table 10**). Among these, *SORCS1* has been associated with Alzheimer’s disease (54) and regulates receptor levels involved in adhesion and neurotransmission (55). Meanwhile, the MTAG analysis with NE uncovered nine additional novel loci: *PTCH2* (1p34.1), *RNF150* (4q31.21), *LINC02143* (5q34), *COA1* (7p13), *TLE1* (9q21.32), *OR1B1* (9q33.2), *ASCL3* (11p15.4), *BATF* (14q24.3), and *EFTUD2* (17q21.31) (**Supplementary Table 11**). Notably, *COA1* and *OR1B1* have been linked to colorectal cancer risk or progression (56, 57), *TLE1* is associated with Crohn’s disease, and both *TLE1* and *BATF* are involved in T-cell-mediated immunity (58, 59). *EFTUD2*, crucial for neuroprotection, has been implicated in mental disorders when dysregulated (60, 61). These findings not only reinforce the brain-gut axis hypothesis in IBS but also unveil potential genetic bridges connecting IBS to cancer risk and immune mechanisms, further corroborating insights from prior TWAS analyses.

### 3.5 Conditional false discovery rate validates core gut-brain pleiotropic hubs and reveals novel IBS-associated loci

To further validate and refine the genetic overlap between IBS and psychiatric traits suggested by MTAG-identified pleiotropic loci, we applied condFDR analysis. This approach identified 57 independent loci, 52 of which were novel compared to the original IBS-UKB/Bellygenes—though most had been reported in subsequent joint IBS studies. The condFDR results strongly confirmed core pleiotropic hubs identified by MTAG, with key loci such as *CCNH* (5q14.3), *LRFN5* (14q21.1), and *NCAM1* (rs7106434) showing highly significant conjoint false discovery rates (conjFDR) (**Supplementary Tables 14**-**17**). Notably, the *NCAM1* locus was further supported by proxy SNPs (rs10891490, rs2186710), reinforcing its role as a key genetic nexus in the gut-brain axis (43). Additional novel loci consistently identified by both methods—including *RERE* (1p36.23), *SLC4A10* (2q24.2), and *SETD5* (3p25.3)—highlight their involvement in neuronal development and synaptic function. The condFDR analysis also revealed novel IBS-associated loci such as *SLC35D1* (1p31.3), *LSAMP* (3q13.31), *COA1* (7p13), and *TLE1* (9q21.32). Among these, *SLC35D1*, *COA1*, and *TLE1* corroborate previous MTAG findings. Notably, LSAMP—previously linked with ulcerative colitis in those of African genetic ancestry (62) and linked to psychiatric traits including bipolar disorder and suicidal behavior (63, 64)—may suggest a potential role in gut-brain axis mechanisms. In contrast to these robust findings, while AN exhibited a significant positive genetic correlation with IBS in LDSC, subsequent condFDR analysis identified only one shared locus (lead SNP: rs750350), a signal already reported in prior IBS GWAS.

### 3.6 Novel genetic loci for IBS show multi-tissue regulation across the gut-brain axis

#### 3.6.1 Tissue-specific expression analysis using GTEx

To elucidate tissue-specific effects of the novel loci identified via MTAG and condFDR, we conducted a comprehensive GTEx screen. We found that rs72679015 (1p31.3) is a trans-eQTL for *DYNLT5* (also known as *TCTEX1D1*) in the brain (Brain-Cerebellum; Brain-Cortex) and colon (Colon-Transverse), with negative normalized effect sizes (NES < 0). *DYNLT5* has been implicated as a depression risk gene (65). At the same locus, we observed a cis-eQTL for *SGIP1* in the brain (Brain-Spinal cord, cervical c-1) and colon (Colon-Transverse), with positive effects (NES > 0). SGIP1 participates in mood and nociception regulation (66) and is upregulated in the intestinal mucosa of Crohn’s disease patients (67). We also identified rs67873618 (7p13) as a cis-eQTL for *COA1* across multiple brain regions (e.g., Brain-Spinal cord, cervical c-1; Brain-Frontal Cortex, BA9) and in Colon - Sigmoid. rs4910157 (11p15.4) acts as a trans-eQTL for *TMEM9B-AS1* across several brain regions (e.g., Brain-Caudate, basal ganglia; Brain-Hypothalamus) and in the colon (Colon-Transverse; Colon-Sigmoid), and as a cis-eQTL for TRIM66 in the brain (Brain-Hippocampus) and colon (Colon-Transverse; Colon-Sigmoid). Finally, rs916888 (17q21.31) is a cis-eQTL for multiple genes—including *KANSL1*, *KANSL1-AS1*, *LRRC37A*, *LRRC37A2*, *MAPK8IP1P1*, *MAPT*, and *WNT3*—in brain regions (e.g., Brain-Anterior cingulate cortex, BA24; Brain-Frontal Cortex, BA9) and colon (Colon-Sigmoid; Colon-Transverse) (**Supplementary Table 18**).

#### 3.6.2 Single-cell eQTL analyses

Single-cell eQTL analyses further resolved regulatory mechanisms of these meta-analysis-identified loci within specific cell types and states, underscoring their central role in the gut-brain axis. Key findings include: rs72679015 regulating *TCTEX1D1* in rotenone-stressed ependymal-like cells and excitatory neurons; rs67873618 bidirectionally modulating *BLVRA*, *COA1*, and *STK17A* during naïve T-cell activation, dopaminergic neuron development, and immune stimulation (e.g., interferon response); rs4910157 functioning as an immune regulatory hub by broadly influencing *AKIP1* expression in Th cells, Treg cells, and B cells, and mediating *TMEM9B/TMEM9B-AS1* responses to viral challenge; and rs916888 (17q21.31) operating as a “super-eQTL,” exerting strong effects on genes such as *KANSL1* and *LRRC37A2* across major CNS cell types (e.g., oligodendrocytes, microglia, neurons) as well as immune cells (**Supplementary Table 19**). Together, these results anchor genetic risk at the intersection of neurodevelopment, immune response, and environmental stressors (infection/toxins), reinforcing neuro-immune crosstalk as a key mechanism underlying gut-brain axis disorders and providing cellular-resolution genetic evidence to guide functional follow-up and target discovery.

#### 3.6.3 mQTL analysis

mQTL analysis revealed that these novel MTAG loci are also potent regulators of DNA methylation, with extensive and strong effects. Each SNP was significantly associated with methylation levels at numerous CpG sites (p-values ranging from 10–^8^ to beyond 10^-200^). Notably, rs916888 (17q21.31) showed particularly prominent effects, associating with more than 60 CpGs and very large effect sizes (e.g., cg24801230, β = −1.37) (**Supplementary Table 20**). These mQTLs occur not only within genes but also across upstream and downstream distal regions, forming complex clusters of methylation quantitative trait loci. This pattern suggests that these variants act by systematically reshaping the local epigenomic landscape. Their strong methylation effects likely represent upstream mechanisms that drive the observed eQTLs and ultimately influence gut-brain axis phenotypes.

## 4 Discussion

This study provides a comprehensive genetic and functional annotation map of irritable bowel syndrome (IBS), integrating the largest available GWAS meta-analysis—combining the IBS-UKB/Bellygenes cohort and two MVP-based analyses—with transcriptomic, epigenomic, and cross-trait data. By systematically layering multi-tissue and single-cell eQTLs, mQTLs, TWAS, and multi-trait inference (MTAG and condFDR), we delineate a multi-level genetic framework for IBS along the brain-gut axis.

The meta-analysis uncovered multiple novel loci beyond previous single-cohort studies. Notably, rs1580180 (MVP1) and rs4738680 (MVP2)—despite being based on different phenotyping criteria within MVP—converged within the same LD block (R² = 0.96), underscoring signal robustness across heterogeneous definitions. This dual-phenotype strategy highlights that core genetic risks for IBS are consistent whether captured through patient surveys or clinical diagnosis records, while also accounting for the phenotypic heterogeneity inherent in complex gut-brain axis disorders. Rs20551, mapped to the MEI1/XPNPEP3 region, was replicated across both meta-analyses and downstream TWAS, suggesting biological importance. Several of these loci also overlapped with or were in strong LD with previously reported signals from MTAG and condFDR analyses (13–15), reflecting expanded discovery power rather than chance findings.

Integration of multi-tissue eQTL data from GTEx showed consistent regulatory effects across colonic and brain tissues. For instance, rs72679015 was found to influence DYNLT5 and SGIP1 expression in both cerebellar and colonic tissues—genes previously implicated in depression and visceral pain (65, 66). Similarly, rs67873618 modulated COA1 expression in the frontal cortex and sigmoid colon, suggesting links between mitochondrial function and visceral symptomatology.

Single-cell eQTL analyses further highlighted cell-type-specific mechanisms. Rs4910157, for example, regulated AKIP1 and TMEM9B-AS1 across CD4^+^ T cells and microglia, providing a mechanistic bridge between immune activation and neuronal signaling. Rs916888 at 17q21.31 emerged as a potential regulatory hub, with widespread cis-eQTL effects on KANSL1, LRRC37A2, MAPT, and WNT3 across major CNS and immune cell types. It also displayed extensive mQTL associations, affecting over 60 CpG sites and suggesting coordinated epigenetic remodeling as an upstream driver of gene expression and cellular phenotypes.

Despite modest SNP-based heritability, We observed strong genome-wide genetic correlations between IBS and several psychiatric traits, including major depressive disorder (MDD), neuroticism (NE), obsessive–compulsive disorder (OCD), post-traumatic stress disorder (PTSD), and attention-deficit/hyperactivity disorder (ADHD) (13, 14). These correlations remained consistent across datasets and reflect shared neurodevelopmental and immunological architecture. This was further substantiated by local heritability and MTAG/condFDR analyses, which highlighted pleiotropic loci such as NCAM1, SETD5, and RERE, linking neuronal adhesion, synaptic plasticity, and psychiatric vulnerability.

Functional annotations across TWAS and QTLs collectively implicate coordinated dysregulation of metabolic (e.g., SIRT3, COA1), membrane lipid (e.g., AGMO), and immune signaling (e.g., PDCD1LG2, IRF7) pathways. These span colon, brain, and blood tissues, suggesting that IBS pathophysiology arises from the interaction of neurotransmitter signaling, immune modulation, and epithelial integrity—rather than being limited to gastrointestinal dysfunction.

Our study leverages the largest and most comprehensive integrated IBS GWAS datasets to date—including UK Biobank, the Bellygenes initiative, and the recent 2024 Million Veteran Program cohorts—which substantially increases statistical power for identifying novel risk loci and enables a more refined characterization of the genetic architecture of IBS. Through systematic integration of multi-omics data, we further provide a high-resolution functional map of the gut–brain axis. Despite the modest heritability of IBS, our analyses reveal strong and consistent genetic correlations with multiple psychiatric disorders, reinforcing its biological connection to the gut–brain axis. Moreover, integrated analyses incorporating tissue-level and cell-type-specific regulatory annotations highlight convergent patterns of genetic regulation across the colon, brain, and immune system, suggesting shared biological mechanisms underlying disease susceptibility. A notable limitation of this study is that the analyses were restricted to individuals of European genetically inferred ancestry, which may limit the generalizability of the findings to other populations and underscores the need for future cross-ancestry genetic studies. Collectively, these results provide a more comprehensive genetic framework for IBS and offer insights that may guide downstream functional investigations and the development of targeted therapeutic strategies.

## Data Availability

The original contributions presented in the study are included in the article/**Supplementary Material**. Further inquiries can be directed to the corresponding authors.
